# Reduced expression of *FOXE1* in differentiated thyroid cancer, the contribution of CPG methylation, and their clinical relevance

**DOI:** 10.3389/fendo.2024.1454349

**Published:** 2024-11-11

**Authors:** Erika Urbano De Lima, Filipe Ferreira Dos Santos, Igor Campos Da Silva, Cláudio Rogério Alves De Lima, Vitoria Sousa Frutuoso, Gustavo Felisola Caso, Paloma Ramos De Oliveira, Ana Karina Bezerra, Janete Maria Cerutti, Rodrigo Esaki Tamura, Helton Estrela Ramos, Ileana Gabriela Sanchez de Rubio

**Affiliations:** ^1^ Laboratório de Ciências Moleculares da Tireoide (LCMT) e Laboratório de Biologia Molecular do Câncer (LBMC), Universidade Federal de São Paulo (UNIFESP), São Paulo, Brazil; ^2^ Centro de Oncologia Molecular (MOC), Hospital Sírio-Libanês - Instituto de Ensino e Pesquisa (HSL-IEP), São Paulo, Brazil; ^3^ Department of Biochemistry, Chemistry Institute (IQ), Universidade de São Paulo (USP), São Paulo, Brazil; ^4^ Departamento de Cirurgia de Cabeça e Pescoço, Monte Tabor – Hospital São Rafael, Salvador, Brazil; ^5^ Universidade de Fortaleza – Unifor, Fortaleza, Brazil; ^6^ Laboratório de Bases Genéticas dos Tumores da Tiroide, Departamento de Morfologia e Genética Universidade Federal de São Paulo (UNIFESP), São Paulo, Brazil; ^7^ Departamento de Ciências Biológicas, Universidade Federal de São Paulo (UNIFESP), São Paulo, Brazil; ^8^ Laboratório de Estudos da Tireoide, Departamento de Bioregulação, Universidade Federal da Bahia (UFBA), Salvador, Brazil

**Keywords:** FOXE1, expression, DNA methylation, differentiated thyroid cancer, aggressiveness

## Abstract

**Introduction:**

Forkhead box E1 (*FOXE1*) is a transcription factor with a crucial role in thyroid morphogenesis and differentiation. Promoter hypermethylation downregulates *FOXE1* expression in different tumor types; nevertheless, its expression and relationship with methylation status in differentiated thyroid cancer (DTC) remain unclear.

**Methods:**

A total of 33 pairs of matched samples of PTC tumors and non-tumors were included. Tumor cell cultures were treated with either 5-Aza-2′-deoxycytidine demethylating agent or dimethyl sulfoxide (DMSO). A real-time polymerase chain reaction (RT-PCR) and Western blotting were performed to assess FOXE1 expression. The methylation status was quantified using bisulfite sequencing. A luciferase gene assay was used to determine CpG-island functionality. Gene expression and promoter methylation of FOXE1 and FOXE1-regulated genes were also analyzed with data from The Cancer Genome Atlas (TCGA) thyroid samples.

**Results:**

After demethylating treatment, increased *FOXE1* mRNA was observed concomitantly with reduced promoter methylation of CpGisland2. A negative correlation between mRNA downregulation and an increased methylation level of CpGisland2 was observed in tumors. Diminished protein expression was also detected in some DTC cell lines and in some tumor samples, suggesting the involvement of post-transcriptional regulatory mechanisms. CPGisland2 was proved to be an enhancer. TCGA data analysis showed low *FOXE1* mRNA expression in tumors with a negative correlation with methylation status and a positive correlation with the expression of most of its target genes. Reduced *FOXE1* expression, accompanied by a high methylation level, was associated with PTC aggressiveness (tall cell variant, advanced extra thyroid extension, T4 American Joint Committee on Cancer (AJCC) classification), age at diagnosis (over 45 years old), and presence of a *BRAFV600E* mutation.

**Conclusion:**

*FOXE1* mRNA was downregulated in DTC compared with non-tumors, followed by high CpGisland methylation. A coupling of low mRNA expression and high methylation status was related to characteristics of aggressiveness in DTC tumors.

## 1 Introduction

Thyroid cancer is one of the most common endocrine malignancies worldwide. Differentiated thyroid cancer (DTC) accounts for approximately 90% of all thyroid cancer cases, presenting with a slow disease course and a favorable prognosis in most cases. However, an increase in the incidence and mortality rates of advanced-stage papillary thyroid cancer (PTC) has been observed in recent years (1). A large number of genetic and epigenetic events have already been described in the pathogenesis of the disease, such as the aberrant activation of the metabolic pathways associated with Mitogen-activated protein kinase (MAPK), Phosphoinositide 3-kinases/Protein kinase B (PI3K/AKT) and Isocitrate dehydrogenase 1 (IDH1) (2). Nevertheless, the etiology of DTC is still not fully understood.

The *FOXE1* gene (HGNC 3806) encodes a specific thyroid transcription factor that plays a crucial role in thyroid morphogenesis, development, growth, and differentiation (3). *FOXE1* regulates the transcription of thyroglobulin (*TG*), thyroperoxidase (*TPO*), *NKX2.1*, *PAX8*, Sodium/Iodine Symporter (*NIS*), and *DUOX2*, genes required for hormone synthesis (4, 5), and also regulates PDGFA (6) and ZEB1 in thyroid cancer (7). Germline *FOXE1* mutations were associated with Bamforth–Lazarus syndrome (8), whereas rare somatic mutations were associated with PTC and goiter (9). Functional analysis of a novel mutation identified in one sporadic and one familial PTC suggested that the variant promoted cell proliferation and migration and may be involved in tumorigenesis (10). Previous reports and data from population databanks have shown that the presence of polymorphisms such as rs965513 and rs1867277 lead to increased susceptibility of developing DTC (11–13).

In thyroid cancer, there is no consensus on the expression of *FOXE1*, which has either been upregulated, downregulated, or had similar expression patterns in tumor and non-tumor samples (6, 22, 23). Aberrant methylation along with aberrant transcriptional gene expression has been identified in almost all types of cancer. Low *FOXE1* expression in parallel with promoter hypermethylation was observed in colorectal, salivary gland, skin, and other types of cancer (14–17), and it was proposed that methylation of *FOXE1* could be a promising marker for cancer detection (18, 19). Combined data from gene expression, polymorphisms, enhancer regions, and methylation have suggested that *FOXE1* could be a novel tumor suppressor (6, 18, 20, 21). In addition, a few studies have investigated the methylation status of *FOXE1* in thyroid tissue. Abu-Khudir et al. (24) observed a reduction in the methylation of two CpG sites of the *FOXE1* promoter that modify its expression in non-tumor thyroid samples. Therefore, in the present study, we investigated both the expression and the promoter methylation status of the *FOXE1* gene in a series of DTC tissues and thyroid tumor cell lines, and thyroid data from The Cancer Genome Atlas (TCGA) database, as well as their clinical relevance. With this approach, we contribute novel data on the reduced expression of *FOXE1* in DTC, the involvement of DNA methylation in *FOXE1* expression, and the association of reduced expression and high methylation with characteristics of aggressiveness.

## 2 Patients and methods

### 2.1 Clinical specimens

The series consists of 33 paired samples, either PTC tumor (T) or non-tumor (NT). Clinical data and the pathological diagnoses of all patients are shown in **Supplementary Table S1** (24–27). The study was conducted under the approval of the Ethics Committee for Research Projects at the Federal University of Bahia and all patients signed an informed consent form.

### 2.2 Cell treatment with 5aza demethylating agent

Thyroid tumor cell lines FTC236 (FTC: follicular thyroid cancer; ECACC General collection, catalog no. 06030202), FTC238 (ECACC General collection, catalog no. 94060902), WRO (metastatic thyroid FTC), and NPA (melanoma cell line) were treated with 5aza-2′-deoxycytidine (5aza) (Sigma-Aldrich, USA). Cells (5.0 × 10^5^) were grown at 37°C in 5% CO_2_ for 4 consecutive days in adequate culture medium with 15 μM 5aza diluted in dimethyl sulfoxide (DMSO) (Sigma-Aldrich, USA) or DMSO alone as a control as previously reported (27–29). All assays were performed in triplicate. After treatment, total RNA, genomic DNA, and protein were extracted for subsequent analysis.

### 2.3 Quantitative real-time polymerase chain reaction

Total RNA was isolated using the Trizol^®^ method and reverse-transcribed with a SuperScript III reverse transcriptase kit (Life Technologies, USA). Quantification was performed with a nanoespectrophotometer (KASVI, Brazil). The quantitative real-time polymerase chain reaction (qRT-PCR) was carried out using Platinum SYBR Green Master Mix (Thermo Fisher Scientific, USA) in a 7500 Real-Time PCR System (Life Technologies, USA), and normalized with *S8* gene expression. PCR primers and conditions are summarized in **Supplementary Table S2**.

### 2.4 CpG island *in silico* identification

The gene sequence information was obtained from Ensembl Gene ID ENSG00000178919. The 10,000 bp upstream to 1000 bp downstream sequence of the *FOXE1* translational start codon, which included exon 1 of the *FOXE1* gene, was investigated for the detection of CpG islands using strict software, namely, the “CpG Island Search” 1.3 cpgi130.pl version (http://cpgislands.usc.edu), “CpGcluster” (http://bioinfo2.ugr.es/CpGcluster), the “RepeatMasker” open version 3.08 (http://www.repeatmasker.org), and the “CpG Island Research,” as previously described (28).

### 2.5 Bisulfite sequencing

DNA was purified from untreated and 5aza-treated cells as well as thyroid samples, as previously reported (24, 25, 27, 28, 31). To discriminate the methylated cytosines from the unmethylated, 1 μg of genomic DNA was converted using the EpiTect Bisulfite conversion kit (QIAgen, Germany) according to the manufacturer’s (28) protocols. Bisulfite-converted DNA (100 ng) was used as a template in 25 μL PCR reactions containing 20 pmol of specific primers: 20 mM dNTPs, 100 mM MgCl_2_, 20 mM Tris-HCl, 50 mM KCl, and 3U Platinum Taq DNA polymerase (Life Technologies, USA). Primer sequences and PCR conditions are described in **Supplementary Table S2**. EpiTect Control DNA (Qiagen) was used as a positive control. PCR products were cloned into a pCR2.1-TOPO vector using a TOPO TA Cloning kit (Life Technologies, USA) according to the manufacturer’s protocols. Individual clones from each sample were amplified and sequenced using a BigDye Terminator v3.1 Cycle Sequencing Kit (Life Technologies, USA). The degree of methylation at each CpG site in each region was expressed as a percentage, determined as the ratio of methylated cytosines to total cytosines.

### 2.6 Western blot analysis

Immunoblotting (10% SDS-PAGE) was performed using an anti-α TTF2 antibody (1:1,000; Affinity BioReagents, USA), Tubulin antibody (1:10,000; Sigma-Aldrich), and GAPDH antibody (1:10,000; Sigma-Aldrich). Band densitometry was performed using ImageQuant LAS 4000 (GE Healthcare Life Sciences, USA) and ImageJ software (National Institute of Health, USA).

### 2.7 Methylation and gene expression analysis from TCGA database

**Figure 1:**
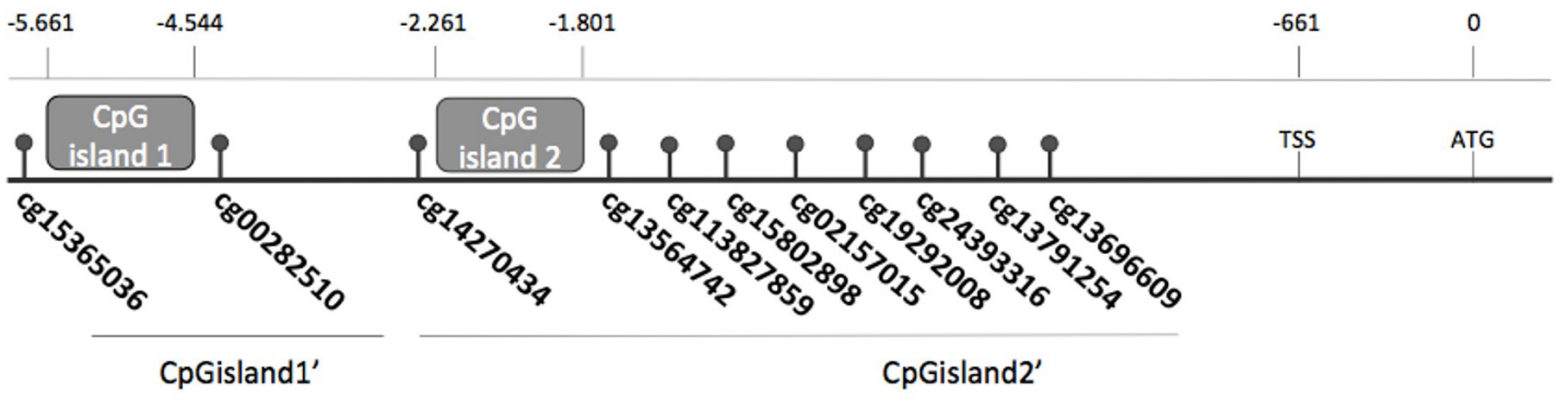
*FOXE1* CpG (cg) sites described in TCGA database indicating the positions of CpGisland1 and CpGisland2 and TCGA sites included in CpGisland1’ and CpGisland2’.

A total 564 samples (508 papillary T and 56 NT tissues) were selected for analysis of the 580 TCGA thyroid samples (THCA) (515 T, 65 NT) (http://cancergenome.nih.gov) (30). Data from samples classified as “Poorly Differentiated Thyroid Cancer” and from patients that received neoadjuvant therapy prior to resection or with incomplete clinical data were not included. The *FOXE1* methylation beta values from TCGA data (level 2) were collected from the Xena Public Data Hubs (https://xena.ucsc.edu/). The CpG sites between cg15365036 and cg13696609 were considered relevant and divided into three regions named CpGisland1’ with 2 CpGs (cg15365036 and cg00282510), CpGisland2’ with 9 CpGs (from cg14270434 to cg13696609), and 2-sites-CpGisland2’ with two CpGs that surround the CpGisland2 (cg15802898 and cg13564742) (**Figure 1**). The methylation value of each island was calculated as the sum of its beta values and a threshold methylation value (CpGisland1’: 0.09996; CpGisland2’: 0.66276 and 2-sites-CpGisland2’: 0.2086; 1.89), which was determined by considering the distribution of the beta values for the NT and T samples as previously described (27) and used to categorize the T samples as hypermethylated. Data for *FOXE1* and FOXE1 target gene expression were also collected from the Xena Public Data Hubs as RSEM values (normalized expectation maximization values) for gene expression (polyA + Illumina Hiseq) (level 3). The clinical data of these patients were downloaded from the Cbioportal for cancer genomics (level 3) (http://www.cbioportal.org/study?id=thca_tcga#clinical) (31). Clinical and pathologic characteristics are presented in **Supplementary Table S3**.

### 2.8 Functional analysis of FOXE1 CpGisland2

The regulatory activity of the region containing CpGisland2 was evaluated using a luciferase reporter assay. The DNA of a thyroid control sample was used in the PCR to amplify the CpGisland2 region with the primers for cloning CpGisland2, containing *Kpn*1 or *Xho*1 restriction sites (**Supplementary Table S2**). The pGL3control (pGL3c) vector and the PCR product were digested with *Kpn*I and *Xho*I restriction enzymes (Invitrogen), following the manufacturer’s protocol. A 10μL ligation reaction mixture containing a 1:10 insert/vector ratio, 1 μL of HiT4 DNA ligase (New England Biolabs, USA), and 1μL of 10X reaction buffer was prepared and incubated for 18 h at 16°C. *Escherichia coli* JM109 was transformed using electroporation and the recombinant plasmid pGL3c-CPG2 was confirmed by PCR and sequencing.

For functional analysis, HEK293 cells (1x10^5^) were cotransfected in a 24-well plate with firefly luciferase plasmid (pGL3c-CPG2 or pGL3c) (2500 ng) and an expression vector of β-galactosidase (600 ng) to normalize the luciferase activity, with the Lipofectamine 3000 Reagent (ThermoFisher Scientific). The experiments were carried out with or without 600 ng of the vectors expressing the transcription factors *PAX8* and *NKX2.1* (28). Nontransfected cells were the negative control of the experiments. The cells were lysed 48 h after the transfections and 30uL of the lysates were used to measure the luciferase activity with a Luciferase Assay System kit (Promega, USA) in a Wallac Victor2 1420 Multilabel Counter (Perkin Elmer, USA). For data normalization, 80uL of ONPG (O-nitrophenyl-beta-D-galactopyranoside) (Sigma-Aldrich) was added to 30uL of the lysates and, after incubation at 37°C for 30 mins, the β-galactosidase was measured at 405 nm using the same equipment.

### 2.9 Statistical analysis

All data were analyzed with SPSS version 22 and GraphPad Prism 5. Results were expressed as mean ± standard error (SEM) or median (minimum-maximum). For the categorical variables, the *χ* ^2^ test or the Fisher’s exact test was used. For the continuous non-parametric variables, Spearman’s correlation and the two-tailed Mann–Whitney or Kruskal–*W*allis tests were performed and receiver operating characteristic (ROC) curve analysis was used to determine the cut-off value for mRNA expression. Fisher’s exact test, the *χ* ^2^ test, the Kruskal–Wallis test, and multivariate logistic regression analysis with Bonferroni corrections were performed to analyze the correlation of methylation status with the clinicopathological parameters of TCGA data. For the luciferase assay, one-way analysis of variance (ANOVA) with Tukey’s multiple comparisons was used. An alpha error of 5% (*p* ¾ 0.05) was considered significant.

## 3 Results

### 3.1 Identification of CpG islands within the *FOXE1* promoter

After *in silico* analysis, we identified two CpG islands. The first one was new and named CpG island1, and comprises a 200 bp sequence with 22 CpG sites and was located between positions −5,661 and −4,527 relative to the ATG site. The second one, a sequence of 460 nucleotides with 24 CpG sites, was referred to as CpGisland2 (positions -2261 to -1801 of the ATG) and has been previously described (24) (**Figure 1**).

### 3.2 *FOXE1* expression and methylation analysis in thyroid carcinoma cell lines

**Figure 2:**
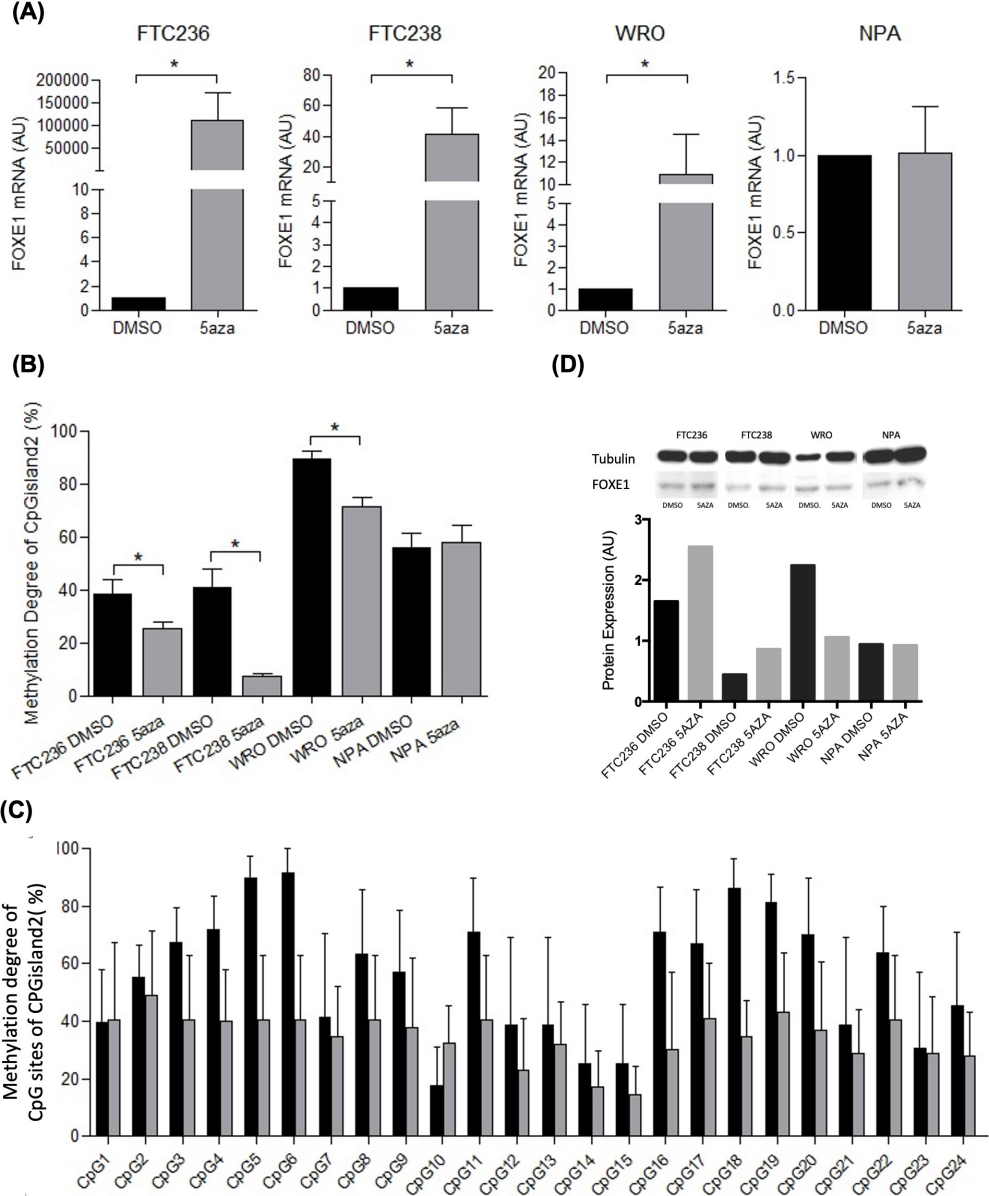
*FOXE1* expression and methylation degree of CpGisland2 in thyroid tumor cell lines (FTC 236, FTC 238, and WRO) and melanoma (NPA) before (DMSO) and after treatment with 5aza. **(A)** Increased *FOXE1* mRNA expression and **(B)** reduced methylation degree (%) in FTC 236, FTC238, and WRO cells after treatment; **(C)** methylation degree (%) of each CpG site in CpGisland2 in FTC 236, FTC 238, and WRO cells; and **(D)** increased FOXE1 protein expression after treatment in FTC236, FTC238, and WRO cells (endogenous control: tubulin). *p < 0.05 (mean ± SEM). Black bar: (DMSO) samples; gray bar: 5aza samples.

FTC 236, FTC 238, WRO, and NPA cell cultures were treated with 5aza to investigate whether demethylation promoted increased expression of *FOXE1* mRNA. The treatment resulted in a significant increase in mRNA expression in FTC 238 [9.58 (0.00031 - 175.55) AU], WRO [15.03 (2.11 - 18.32) AU], and FTC 236 [36148.2 (27.12 - 339958.7) AU] (*P* < 0.05) compared to untreated cells. No effect was observed in NPA [0.678 (0.2662 – 2.012) AU] (**Figure 2A**).

A significantly lower methylation degree of CpGisland2 was observed in FTC236, FTC238, and WRO 5aza-treated cells [30% (0%-40%), 7.69% (0%-15.38%), and 79.16% (33.33%-91.67%), respectively] compared to untreated cells [33.33% (8.33%-75%), 34.61% (0%-100%), and 94.44% (44.44%-100%), respectively] (*P* < 0.05) (**Figure 2B**). The combined analysis of the 22 CpGs in the CpGisland2 of FTC 236, FTC 238, and WRO cells showed a reduction in the methylation degree after treatment of almost all sites, but it was not statistically significant (p>0.05) (**Figure 2C**). In parallel, an increase in FOXE1 protein expression was observed in FTC236 and FTC238 5aza-treated cells compared to untreated cells, while increased expression was observed in the WRO cells and no difference in the NPA cells (**Figure 2D**). The analysis of CpGisland1 showed that the CpGs were methylated to different degrees, but there was no difference in the overall methylation levels between the treated and untreated cell lines (**Supplementary Figure S1)**.

### 3.3 FOXE1 expression and promoter methylation in papillary thyroid carcinoma

**Figure 3:**
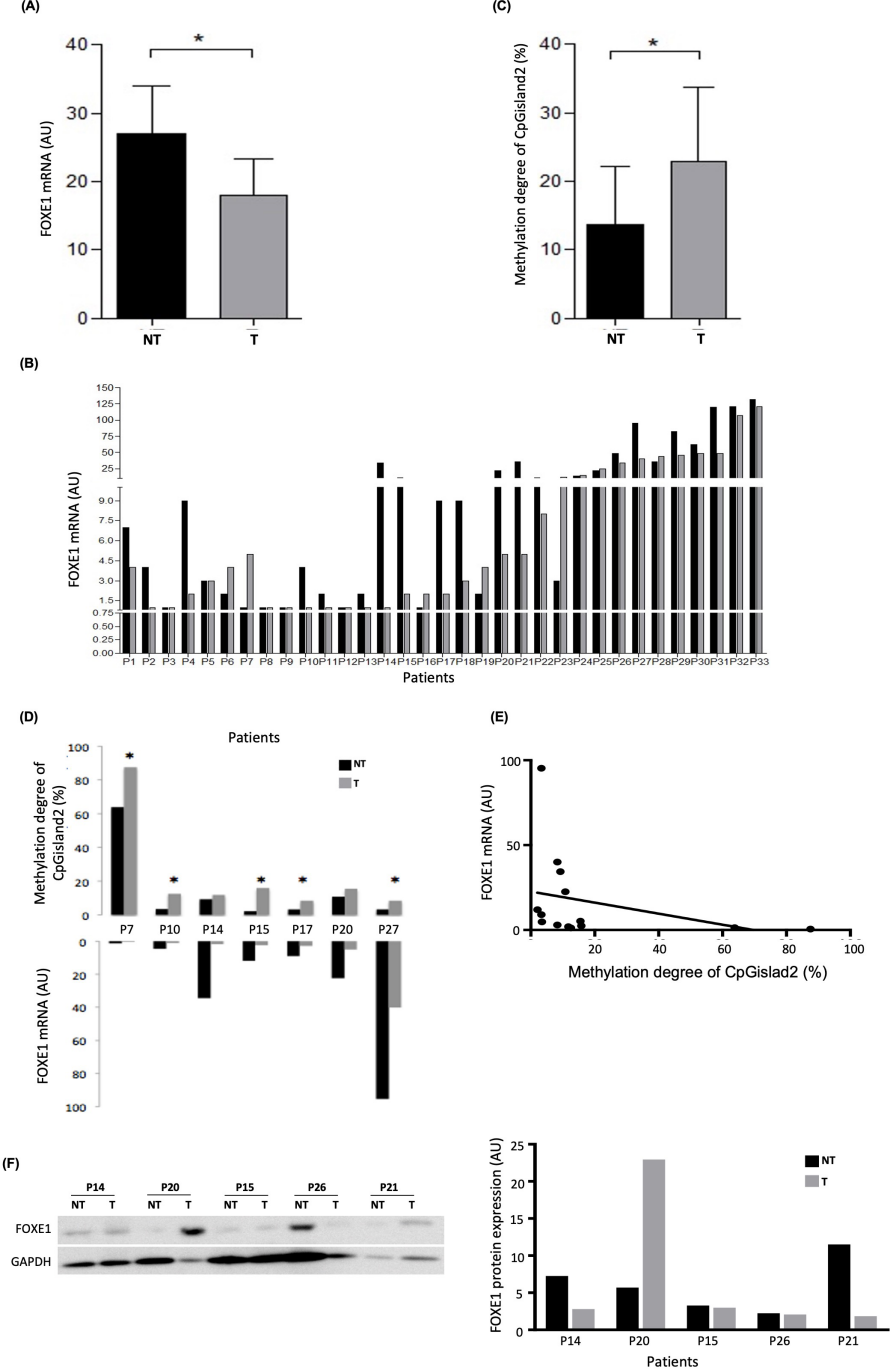
*FOXE1* expression and methylation analysis of the 33 PTC samples pairs (T and NT). **(A)** Reduced *FOXE1* mRNA expression in T compared with NT matched samples; **(B)** 69.7% of the sample pairs showing reduced *FOXE1* expression in T compared to NT; **(C)** high methylation degree (%) of CpGisland2 in T compared to NT matched samples; **(D)** high methylation degree (%) coupled with reduced expression of seven pairs of T and NT matched samples. **(E)** Negative Spearman’s correlation between methylation and *FOXE1* expression (r = -0.698, *p* = 0.003). **(F)** Relative FOXE1 protein expression in five pairs of samples with reduced mRNA expression of T compared to NT (endogenous control: β-actin). *p < 0.05 (mean ± SEM). Black bar: NT samples; gray bar: T samples.

We compared *FOXE1* mRNA levels in T and NT matched samples from 33 patients with PTC. The T samples showed reduced expression [2.89 (0.044 - 107.86) AU] compared with their adjacent NT samples [4.06 (0.072 - 121.75) AU] (*P* = 0.0096) (**Figure 3A**). In total, 22 of the 33 pairs (69.7%) presented downregulated *FOXE1* mRNA expression in the T samples compared to the corresponding NT samples (**Figure 3B**). An empirical mRNA expression cut-off value of 5 AU allowed us to associate higher Bethesda classifications (Bethesda IV, V and VI) with lower mRNA expression (*P* = 0.006).

The methylation analysis of CpGisland1 and CpGisland2 was performed in nine (P7, P9, P10, P14, P15, P17, P20, P22, and P27) and in seven pairs of samples (P7, P10, P14, P15, P17, P20, and P27), respectively. Bisulfite sequencing of the 22 CpG sites of CpGisland2 revealed a higher methylation degree in the T samples [12.5 (8.33 - 87.5) %] compared to the NT samples [3.47 (2.08 - 63.78) %] (*P* = 0.0177) (**Figure 3C**). **Figure 3D** details the results of the individual samples, in which all pairs presented reduced T mRNA expression with increased T methylation levels, and was significant in 71.43% of the cases (*P* < 0.05). There was a negative correlation between the methylation degree of CpGisland2 and mRNA expression when all samples (T and NT samples) were analyzed together (r = -0.698, P = 0.003) (**Figure 3E**). No difference in the methylation degree between the T and NT samples was observed for CpGisland1 (**Supplementary Figure S2**) and in only 44% of the pairs of samples was there an increase in the T methylation degree. There was no correlation between mRNA expression and the methylation degree of CpGisland1. Reduced relative protein expression was observed in the T samples compared to the NT samples in four of 5 pairs (two with very slight reduction) (**Figure 3F**).

### 3.4 FOXE1 expression and methylation analysis using TCGA data

**Figure 4:**
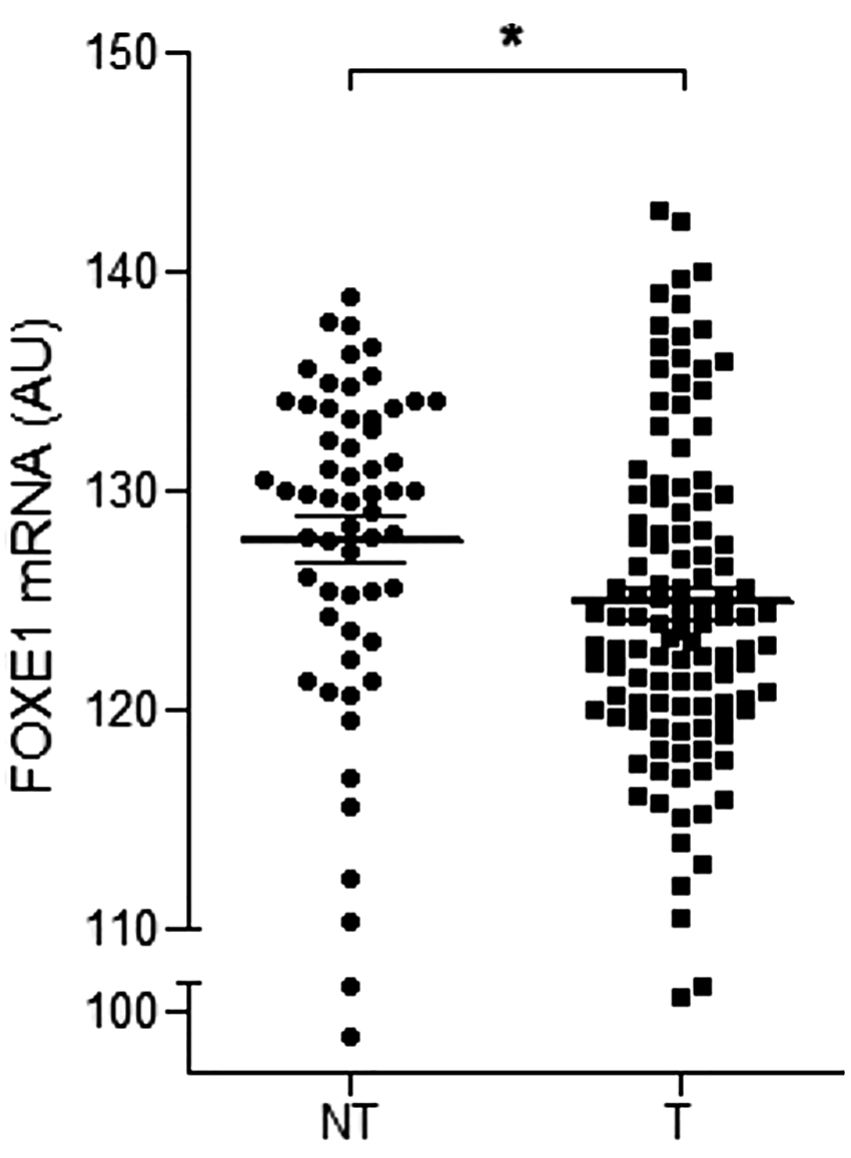
Downregulation of *FOXE1* mRNA expression in thyroid T samples from TCGA dataset compared to NT samples. *p < 0.05 (mean ± SEM).

TCGA data analysis showed *FOXE1* mRNA expression (123511,54 ± 7912,788 AU) was downregulated in the T samples compared to the NT samples (127742.39 ± 8214.723 AU) (*P* < 0.001) (**Figure 4**). Among the PTC subtypes, the tall cell variant presented lower mRNA expression (120030.35 ± 4658.109 AU) compared to the classical (122634.57 ± 7618.959 AU) and follicular variants (127604.16 ± 8023.145 AU) (*P* < 0.001). When considering the clinical characteristics, the T samples with advanced extra thyroid extension exhibited lower *FOXE1* mRNA expression (106590 ± 0) than those with minimal extension (118671.25 ± 5450.599) or without extension (121654.82 ± 6788,95) (*P* = 0.001), and the T samples with a more advanced classification presented lower *FOXE1* mRNA expression (T4 < T3 < T2 < T1 and N1 < N0) (*P* < 0.001).

No difference in the methylation of CpGisland1’, CpGisland2’, and 2-sites-CpGisland2’ was detected between the T and NT samples. However, in the tumor samples, significant negative correlations between *FOXE1* mRNA and the methylation values of the three regions were observed (CpGisland2’ r = -0.164; 2-sites-CpGisland2’ r = 0.218; CpGisland1’ r = -0.095, *P* < 0.04), while no correlation was observed in the non-tumor samples. Considering the clinical characteristics, among the PTC subtypes, the tall cell variant showed a higher methylation of CpGisland2’ (0.673637 ± 0.2435934 AU) than the classical (0.608464 ± 0.2789129 AU) (*P* < 0.001) and follicular variants (0.537720 ± 0.2357689 AU) (*P* < 0.001). Samples from older patients at diagnosis exhibited a high methylation degree in CpGisland2’ (>45 years old: 0.615680 ± 0.2916553 AU; <45 years old: 0.577995 ± 0.2398365 AU; *P* = 0.016). We observed a decrease in the methylation levels of CpGisland1’ and 2-sites-CpGisland2’ in the primary tumor samples (0.112480 ± 0.0416035 AU and 0.1848450 ± 0.0988664) compared to metastasis (0.1507000 ± 0.0533801 AU and 0.253413 ± 0.1108841 AU) (*P* = 0.042 and *P* = 0.046, respectively). When applying the threshold cut-off value, hypermethylation of CpGisland1’ was associated with bilateral tumors (56/87, 64.4%) (unilateral: 214/416, 51.4%, *P* = 0.033) and hypermethylation of CpGisland2’ and 2-sites-CpGisland2’ was associated with the tall cell PTC variant (*P* = 0.014 and *P* = 0.039, respectively).

An association between the presence of the *BRAF* ^V600E^ mutation, mRNA *FOXE1* expression, and methylation was detected, where the *BRAF* ^V600E+^ tumors had lower expression (121727.54 ± 5419.814 AU) and higher methylation (0.615242 ± 0.26681367 AU) of CpGisland2’ compared to the *BRAF* ^V600E-^ tumor samples (mRNA expression: 124985.91 ± 9281.498 AU and CpGisland2’ methylation: 0.584073 ± 0.2705702 AU) (*P* < 0.05).

### 3.5 Expression of *FOXE1* target genes

To understand the relevance of reduced mRNA *FOXE1* expression in PTC, the expression of several *FOXE1* target genes, i.e., *PAX8*, *TG, TPO, DUOX2, NIS, NKX2.1, PDGFA, and ZEB1* (6, 7, 32) and their correlation with *FOXE1* mRNA expression was investigated. The analysis of the TCGA data showed reduced expression of *PAX8*, *TG*, *TPO*, *NIS* and *PDGFA* in T compared to NT samples (*P* < 0.05 each), while no differences of the expression of *NKX2.1*, *DUOX2* and *ZEB1* were detected (**Supplementary Figure S3**). Moreover, in the T samples, positive correlations between *FOXE1* expression and *PAX8*, *TG*, *TPO*, *DUOX2*, *NIS*, and *ZEB1* were observed, but PDGFA showed a negative correlation (*P* < 0.01 each). In the NT samples, only positive correlations were observed with *PAX8*, *TG*, *TPO*, *DUOX2*, and NKX2.1. Interestingly, in the T samples, a negative correlation was observed between the methylation state of the FOXE1 CPGisland 2’ and *PAX8*, *TG, DUOX2*, and *NKX2.1*, while a positive correlation was found with *NIS* and *PDGFA.* In the NT samples, a negative correlation was detected with *PAX8*, *DUOX2*, and *NKX2.1* and a positive correlation with *PDGFA* (**Supplementary Table S4**). In the patient samples, the *TG* and PAX8 expression was reduced in the T samples compared with the NT samples (**Supplementary Figure S4**), without significance, and no correlation with FOXE1 expression was observed, probably due to the reduced number of samples.

### 3.6 Functional analysis of FOXE1 CpGisland2

**Figure 5:**
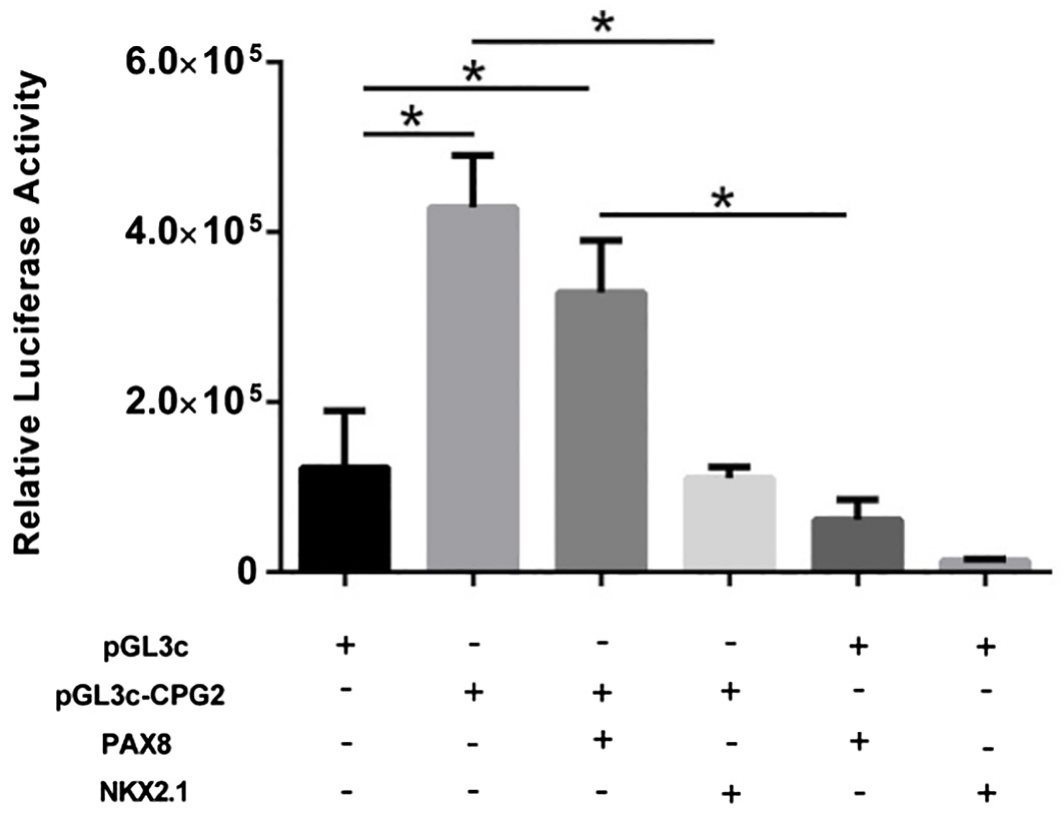
Relative luciferase expression in HEK293 cells transfected with pGL3c or pGL3c-CPG2 in the presence or absence of plasmids expressing the transcription factors NKX2.1 and PAX8 was evaluated to assess the regulatory activity of FOXE1 CpG-island2 (*p < 0.005).

To investigate whether the CPGisland2 was able to regulate the activity of a promoter and whether the CPGisland2 activity could be modulated by the transcription factors *PAX8* and *NKX2.1*, a luciferase reporter assay with pGL3control-derived vectors was conducted. Cells transfected with pGL3c-CpG2 showed a significantly higher expression of luciferase compared with the pGL3c-transfected cells (*P* < 0.05), confirming the regulatory activity of the CPGisland2. The expression of *PAX8* and *NKX2.1* did not increase CPGisland2 activity. Interestingly, the expression of *NKX2.1* notably reduced luciferase activity, suggesting repressor activity through the pGL3c promoter which was not overcome by the presence of CPGisland2. In contrast, CPG2island2 was able to restore luciferase activity in the presence of *PAX8* (**Figure 5**).

## 4 Discussion

In the last few decades, studies have attempted to understand the role of the transcription factor *FOXE1* in DTC by identifying mutations in sporadic or familial cases (9, 10) and polymorphisms associated with increased predisposition (11, 12), but no consensus has been reached on *FOXE1* gene expression in DTC (6, 22, 23). However, a few authors have investigated *FOXE1* expression and its epigenetic regulation in thyroid cancer. Thus, in this study, we sought to better understand *FOXE1* gene expression in DTC in parallel with the involvement of DNA methylation and the clinical relevance of both parameters.

Initially, we performed an *in silico* analysis in a large sequence upstream *FOXE1* gene with strict criteria and identified the new GpGisland1 and the known CpGisland2 (24) (**Figure 1**). Next, we investigated *FOXE1* expression in cell lines and the methylation status of both CpG islands. To our knowledge, this is the first study investigating these two CPG islands in thyroid cancer. Previously, CPG2 was only investigated in ectopic thyroids and leucocytes (24). We observed increased mRNA expression in the three DTC cell lines (FTC 236, FTC 238, and WRO) concomitantly with a low methylation degree of CpGisland2 and an increased protein expression in two of them after the demethylation treatment, indicating a crosstalk between *FOXE1* methylation, mRNA, and protein expression (**Figure 2**). The non-concordance of mRNA and protein levels in WRO suggested the involvement of post-transcriptional regulatory mechanisms (7). It has been shown that microRNAs regulate FOX protein expression (33) as miR-524-5p targets FOXE1, suppressing PTC progression (34). In addition, other events may be considered such as RNA modifications (35) and LncRNA activity (36, 37). In the melanoma (NPA) cell line, no changes were observed in *FOXE1* mRNA, protein expression, and methylation degree after treatment, as we had already observed for the *NIS* and *ABI3* genes (28, 31), probably due to the duration of the treatment or reduced activity of the 5aza demethylating agent in this cell type. We then investigated *FOXE1* mRNA expression in the our patients’ samples (**Figure 3**). We observed reduced *FOXE1* mRNA expression in tumors when compared to non-tumor samples (**Figure 2**), in accordance with *FOXE1* expression in the TCGA dataset (**Figure 3**). Protein expression in the tumors was downregulated in four of the five investigated paired samples (two were slightly reduced), once more suggesting that other regulatory mechanisms play a role in FOXE1 protein synthesis (33) and need further investigation.

*FOXE1* expression has been studied in different tumor types: *FOXE1* downregulation was observed in salivary gland, breast, colon, and skin tumors (15, 16, 18), upregulation was described in basal cell carcinoma, liver, and airway tumors (32–34), and, in pancreatic tumors, both increased and decreased *FOXE1* expression was reported (17, 35, 40). In the thyroid, previous studies suggested oncogene-like behavior for FOXE1, a correlation between the level of *FOXE1* expression and thyroid tumor differentiation status (41, 42) and interference with the *p53* pathway was observed. However, suppression activity of FOXE1 in the early stages of PTC has also been proposed (6). Besides that, transcriptional *FOXE1* expression was significantly downregulated in poorly differentiated thyroid carcinoma and absent in anaplastic thyroid cancer (43–46). Similar results were obtained in a series of thyroid cell lines, where the expression was higher in non-tumor control and PTC than in FTC cells, with the lowest values in the ATC cell lines (7, 9). High *FOXE1* expression was detected in follicular adenomas compared to FTC, as well as in Graves’ disease samples compared to PTC cases (38, 39, 47). Likewise, the overexpression of cytoplasmatic and the downexpression of nuclear *FOXE1* were observed in PTC compared with surrounding NT regions (15). Yet the same group showed that *FOXE1* overexpression in mouse thyroids developed multinodular goiter but not cancer (48). It has to be mentioned that FOXE1 participates in a complex transcriptional network of regulation in thyroid follicular cells (42, 49). Thus, understanding the activity of this transcription factor in thyroid cancer is challenging.

Regarding the involvement of methylation in *FOXE1* expression, in our cohort of DTC samples, we observed low *FOXE1* expression in tumors compared to non-tumor matched samples along with a high *FOXE1* methylation degree of CpGisland2 as well as a significant negative correlation between mRNA expression and methylation. This data suggests that FOXE1 mRNA expression is likely to be modulated by methylation, particularly of CpGisland2. Interestingly, a previous study associated the high methylation of two specific sites (CpG_5_ and CpG_6_) in CpGisland2 in leukocytes, when comparing eutopic and ectopic thyroid tissues, with *FOXE1* expression (24); however, our data did not confirm this association in DTC. Limitations in our study, as well as the other (24), were how to overcome technical constraints in analyzing the CG-rich region of the CpGisland2 of the *FOXE1* gene in all the patient samples in the bisulfite methylation investigation and that TCGA methylation data do not include the CpG sites of the CpG-islands investigated in this study. Even so, the involvement of methylation in *FOXE* expression was corroborated in TCGA data, which showed a significant negative correlation between methylation and *FOXE1* mRNA expression among all the samples (tumor and non-tumor) and in the tumor samples but not in the non-tumor samples. Furthermore, considering a linear regression model, the contribution of the methylation levels in gene expression of the 2-sites-CpGisland2’, which includes the two sites surrounding CpGisland2, was higher (6.3%) than the contribution of the other CpG regions (3.9% and 0.5%), suggesting that the methylation of the region that comprises CpGisland2 may be involved in mRNA regulation. These results may explain the fact that no difference in the methylation status of TCGA regions was observed between the T and NT samples. Nonetheless, the involvement of the methylation of other regions, such as regions downstream of the transcription start site, first exon, intragenic exons and first intron could not be excluded (50), as well as other genetic mechanisms that regulate *FOXE1* expression, such as distant enhancers (51), *FOXE1* mutations, and functional polymorphisms (22).

To advance the understanding of high methylation and reduced mRNA expression in thyroid tumors, the expression of FOXE1 target genes (*TG*, *TPO*, *PAX8*, *NIS*, *DUOX2*, *NKX2.1*, *PDGFA*, and *ZEB1)* was investigated. The analysis of TCGA data showed a moderate positive correlation between the expression of most of the FOXE1 target genes with mRNA *FOXE1* expression and a negative correlation with the methylation levels of CpGisland2’ (**Supplementary Table S4**) in the T and NT samples. An inverse correlation was observed for PDGFA in the T samples. These results suggest the relevance of reduced *FOXE1* expression in thyroid cancer dedifferentiation. Interestingly, conflicting data has been reported previously as Din et al. (6) showed increased *FOXE1* mRNA and protein expression in the early stages of PTC, suggesting tumor suppression activity through the inhibition of proliferation and invasion, probably by negatively regulating *PDGFA* expression. Morrilo-Bernal et al. (7) showed that *FOXE1* expression positively correlates with thyroid cancer differentiation, but the reduction of *FOXE1* expression reduced migration and invasion through the regulation of *ZEB1*.Thus, further studies are necessary to understand the role of *FOXE1* expression in thyroid cancer.

Still, we confirmed the enhancer activity of CPGisland2 using a luciferase gene reporter assay, and PAX8 and NKX2.1 did not modulate its activity, which was in accordance with the lack of these transcription factor sites in this region.

Considering the clinicopathological characteristics in our tumor samples, low mRNA values were associated with an increased risk of malignancy, and TCGA data analysis also showed an association between reduced *FOXE1* mRNA expression and the aggressiveness of PTC subtypes, as previously reported (7), as well as with worse clinicopathological and genetics characteristics, such as extra thyroid invasion, AJCC and TNM classification, and the presence of *BRAF* mutations. Our results are in line with the observed downregulation of FOXE1 gene expression in PTC and no expression in the most advanced and aggressive form of TC (45). However, another report detected an increase of *FOXE1* expression in PTC tumor tissues that correlated with a worse clinical prognosis (14).

In parallel with gene expression, methylation of CpGisland2’ was positively associated with the aggressiveness of PTC subtype, the presence of BRAF mutation, and age at diagnosis (>45 years old). Likewise, the methylation levels of CpGisland1’ and 2-sites-CpGisland2’ were higher in metastases than in primary tumor samples and hypermethylation of CpGisland2’ was associated with bilateral tumors and tall cell variants. Hypermethylation of *FOXE1* was observed in 75% of pancreatic carcinomas, as well as in squamous cell cancer and in salivary gland carcinoma (15, 17). In breast cancer, hypermethylation was identified in the plasma of patients with a more aggressive disease (stage IV) (52) and, in bladder cancer, FOXE1 methylation was suggested as one of the high-grade markers (53). Interestingly, in colorectal cancer, it was shown that high methylation of FOXE1 contributed to the poor prognosis of the patients (16) and could be associated with the observed FOXE1 repression of tumor cell growth and glycolysis through the inhibition of HK2 (23). Besides this, other genes, mechanisms, and risk factors have been linked with DTC aggressiveness (54). We have previously shown that the hypermethylation of the HOPX promoter was associated with a worse prognosis in DTC (27) while the low DNA methylation of three SNP markers was related with recurrent or persistent disease (55). The deregulation of key signal pathways such as the hedgehog pathway was also associated with aggressive PTC (56).

In summary, these results provide novel evidence that *FOXE1* mRNA expression is reduced in DTC, methylation contributes to the regulation of *FOXE1* expression, and the coupling of low mRNA expression and high methylation status can be associated with some characteristics of aggressiveness in DTC tumors. Further investigations involving the methylation status of other *FOXE1* regions or other genetic or epigenetic mechanisms that regulate gene expression, including microRNA, lncRNA, and RNA modifications, are warranted.

## Data Availability

Data supporting the findings of this study are available in cBIOPORTAL at doi: 10.1158/2159-8290.CD-12-0095 and doi: 10.1126/scisignal.2004088 and UCSC XENA at https://doi.org/10.1038/s41587-020-0546-8. The datasets presented in this study can also be found in online repositories. The names of the repository/repositories and accession number(s) can be found below: https://www.cancer.gov/ccg/research/genome-sequencing/tcga, phs00017. Further information is available form the corresponding author on request.
