# Supplementary material for: Reduced expression of FOXE1 in differentiated thyroid cancer, the contribution of CPG methylation, and their clinical relevance
Source: Front Endocrinol (Lausanne). 2024 Nov 11;15:1454349. doi: 10.3389/fendo.2024.1454349 (PMC11586194; doi:10.3389/fendo.2024.1454349)
Supplement: Supplementary file 1 [file DataSheet1.pdf]

Supplementary Data

Figure S1: No difference in the methylation degree (%) of CpGisland1 in untreated (DMSO) and 5aza treated follicular carcinoma cells (FTC236, FTC238 and WRO) and melanoma cells (NPA) (mean ±SEM). Black bar: DMSO samples; gray bar: 5aza samples.

Figure S2 No difference in the methylation degree (%) of CpGisland1 between T and NT samples.

Figure S3: TCGA data analysis of the expression of FOXE1 target genes. Downregulation in T compared to NT of PAX8 TG, TPO, NIS and ZEB1, and upregulation T compared to NT of NKX2.1 and PDGFA * $p<0.0001$, no difference was observed for DUOX2 expression.

Figure S4: Patients' samples analysis of the expression of *TG* and *PAX8.* Low expression in T compared to NT samples without significance ($P > 0.05$).

Figure S1

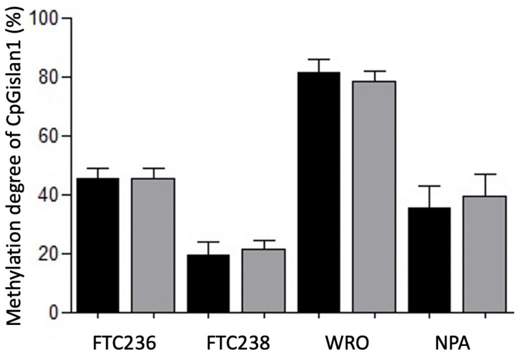

Figure S2

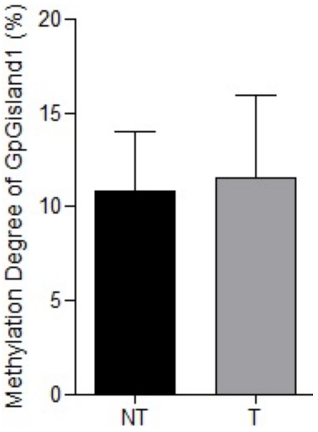

Figure S3

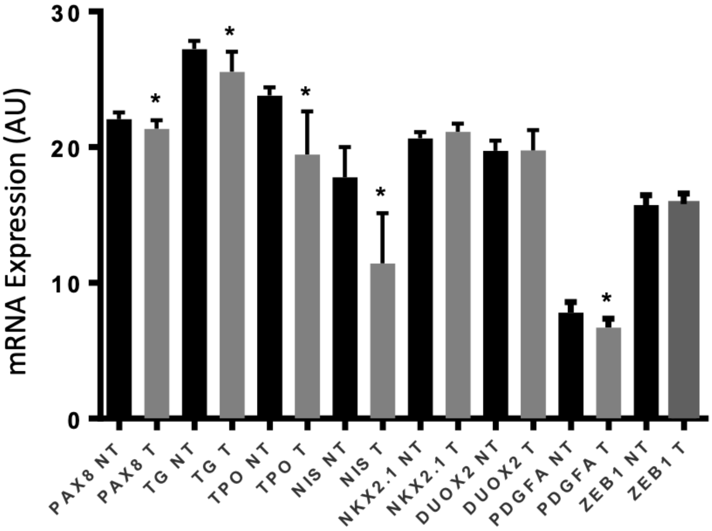

Figure S4

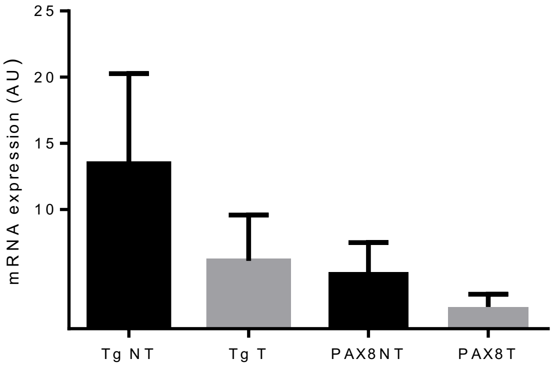

Table S1: Clinical data of the patients.

ID	Age (Y)	Gender	Histology	Variant	Ultrasound Focality	Lymph Node Involvement	T	N	M staging
1	45	M	PTC	Classical	Unifocal	Reactive	T1a	N0	M0
2	30	F	PTC	Classical	Unifocal	Reactive	T1b	N0	M0
3	49	M	PTC	Classical	Unifocal	Pathological	T1a	N1a	M1
4	49	F	PTC	Classical	-	-	-	-	-
5	57	F	PTC	Classical	Multifocal	Reactive	T3	N0	M1
6	38	F	PTC	Classical	Unifocal	Reactive	T3	N0	M0
7	54	M	PTC	Classical	Multifocal	Reactive	T1a	N1a	M1
8	30	F	PTC	Classical	Multifocal	Pathological	T3	N1b	M0
9	64	F	PTC	Classical/ Follicular	Absent	Absent	-	-	-
10	27	F	PTC	Classical	Multifocal	Reactive	T1b	N0	M0
11	55	F	PTC	Follicular	Multifocal	Pathological	T1a	N0	M0
12	27	F	PTC	Classical	Multifocal	Reactive	T1a	N1a	M0
13	-	F	PTC	Classical	Absent	Absent	-	-	-
14	22	F	PTC	Classical	Absent	Absent	-	-	-
15	24	F	PTC	Classical	-	-	-	-	-
16	32	F	PTC	Solid	Unifocal	Pathological	T3	N1a	M0
17	42	F	PTC	Classical	Multifocal	Reactive	T3	N0	M0
18	48	M	PTC	Classical	Multifocal	-	T1a	N0	M0
19	49	F	PTC	Classical	Multifocal	Reactive	T1a	N0	M0
20	31	F	PTC	Tall cell	Absent	Absent	-	-	-
21	40	F	PTC	Diffuse Sclerosing	-	-	-	-	-
22	-	F	PTC	Classical	Absent	Absent	-	-	-
23	59	F	PTC	Clear cell	Absent	Absent	-	-	-
24	-	M	PTC	Oncocytic	Absent	Absent	-	-	-
25	-	F	PTC	Classical	Absent	Absent	-	-	-
26	31	M	PTC	Classical	Absent	Pathological	-	-	-
27	44	F	PTC	Classical	Unifocal	Reactive	T1a	N0	M0
28	-	F	PTC	Classical	Absent	Absent	-	-	-
29	-	F	PTC	Classical	Absent	Absent	-	-	-
30	47	F	PTC	Classical	-	-	-	-	-
31	49	F	PTC	Classical	Absent	Absent	-	-	-
32	44	F	PTC	Classical	-	-	-	-	-
33	-	F	PTC	Follicular	-	-	-	-	-

Table S2: Primer sequences, PCR conditions and product sizes.

Gene/region	Sequence (5'-3')	Application	Annealing (°C)	Product size (pb)
FOXE1	F: GGAGCCCCCTACTCTTTGAC<br>R: TTGTCTGGAAGAGCCTGGAT	Expression	60	100
S8	F: AACAAGAAATACCGTGCCC<br>R: GTACGAACCAGCTCGTTATTAG	Expression	60	100
**CpGIsland1**	F: TAGGGTTTTAGAATTTAATTGAAATG<br>R: ATCACTATCACCCAAAAAACAAAATA	BS	57	200
**CpGIsland2**	F: GTAAGGATTTGGGTTTTGTTT<br>R: AAAACCTCAATATTCTACTCTATTAAA	BS	57	355
**CpGIsland2**	F: CCG**GGTACC**GTTTAGCGAGTGTAAAGCCCC<br>R: CCG**CTCGAG**GCCATTCACTGGGAGATCCA	Cloning	60	500

BS: Bisulfite sequencing, bold sequences indicate restriction sites (*Kpn*1, *Xho*1)

Table S3: Patients characteristics from The Cancer Genome Atlas (TCGA) database

		Number	Percentage
**Diagnosis age**	<45	235	46.3
	≥45	273	53.7
**Gender**	Female	369	72.9
	Male	137	27.1
**Race**	White	333	65.6
	Black or African American	26	5.1
	Asian	51	10
	Not determined	98	19.3
**Ethnicity**	Hispanic or Latin	38	7.5
	No Hispanic or Latin	359	70.7
	Not determined	111	21.9
**Histological type**	Classical	362	71.3
	Follicular	102	20.1
	Tall cells	34	6.7
	Others	8	1.6
	Not determined	2	0.4
**Pathologic T stage**	T1	44	8.7
	T1a	19	3.7
	T1b	80	15.7
	T2	168	33.1
	T3	173	34.1
	T4	9	1.8
	T4a	12	2.4
	Not determined	3	0.6
**Pathologic N stage**	N0	226	44.5
	N1	59	11.6
	N1a	91	17.9
	N1b	80	15.7
	NX	50	9.8
	Not determined	2	0.4
**Pathologic M stage**	M0	281	55.3
	M1	9	1.8
	MX	21	42.3
	Not determined	3	0.6
**AJCC**	I	288	50.9
	II	51	11.4
	III	110	24.6
	IV	2	0.4
	IVa	47	10.5
	IVc	6	1.3
	Not determined	4	0.9
**Disease free status**	Free	445	87.6
	Recurrent or Progressive	49	9.6
	Not determined	14	2.8
**Overall survival**	Living	492	97.2
	Diseased	14	2.8
**New neoplasm event post initial therapy**	Yes	12	2.4
	No	250	49.2
	Not determined	246	48.4
**Surgical margins**	R0	387	76.2
	R1	53	10.4
	R2	4	0.8
	RX	30	5.9
	Not determined	34	6.7

Table S4: Correlation analysis of FOXE1 mRNA expression and CpGisland2'methylarion degree with the expression of genes regulated by FOXE1.

	FOXE1 mRNA		CpGisland2' methylation	
	Tumor samples			
Gene	r	P	r	P
PAX8	0.34	**< 0.0001**	-0.31	**< 0.0001**
TG	0.29	**< 0.0001**	-0.28	**< 0.0001**
TPO	0.30	**< 0.0001**	-0.04	0.1586
DUOX2	0.21	**< 0.0001**	-0.36	**< 0.0001**
NIS	-0.03	0.5032	-0.24	**< 0.0001**
NKX2.1	0.17	**0.0002**	0.09	**0.0181**
PDGFA	-0.23	**<0.0001**	0.11	**0.001**
ZEB1	0.14	**< 0.0001**	-0.36	**< 0.0001**
	Non tumor Samples			
PAX8	0.60	**< 0.0001**	-0.37	**0.008**
TG	0.63	**< 0.0001**	-0.28	**0.051**
TPO	0.46	**0.0005**	-0.03	0.82
NIS	0.31	**0.024**	-0.49	**0.0003**
NKX2.1	0.27	0.051	0.05	0.73
PDGFA	-0.21	0.10	-0.29	**0.035**
ZEB1	0.03	0.80	-0.21	0.17
DUOX2	0.48	**0.0003**	-0.49	**0.0003**

Bold values indicate statistical significance.
